# Kardiovaskuläre Folgen des Rauchens: Radiologische Tour d’Horizon

**DOI:** 10.1007/s00117-022-01027-1

**Published:** 2022-06-20

**Authors:** Mathias Pamminger, Agnes Mayr

**Affiliations:** Universitätsklinik für Radiologie, Medizinische Universität Innsbruck, Anichstr. 35, 6020 Innsbruck, Österreich

**Keywords:** Raucherentwöhnung, Herz-Kreislauf-System, Arteriosklerose, Aufklärung, Prävention, Smoking cessation, Cardiovascular system, Atherosclerosis, Patient education, Prevention

## Abstract

**Klinisches Problem:**

Rauchen beeinflusst das kardiovaskuläre System des Körpers. Primär führt es entweder zu atheromatösen Plaques mit potenzieller Gefäßstenosierung oder zu aneurysmatischen Gefäßveränderungen mit potenzieller Rupturgefahr.

**Radiologische Standardverfahren:**

Je nach Lokalisation ermöglicht die Sonographie eine initiale Einschätzung der Veränderungen. Eine Angiographie in Kombination mit Computertomographie (CT) oder Magnetresonanztomographie (MRT) ermöglicht die weiterführende Beurteilung und ggf. Therapieplanung. Ohne klinische Symptomatik wird bei Rauchern ohne sonstige Risikofaktoren oder Komorbiditäten keine bildgebende Diagnostik lediglich aufgrund des Rauchens empfohlen.

**Methodische Innovationen:**

Aktuelle Leitlinien der entsprechenden Pathologien erkennen das Rauchen einstimmig als modifizierbaren Risikofaktor für kardiovaskuläre Erkrankungen an, weshalb stets eine Raucherentwöhnung als erster Schritt zur Prävention sekundärer Akutereignisse empfohlen wird. Bei Verdacht auf ein chronisches Koronarsyndrom erhöht das Rauchen die klinische Wahrscheinlichkeit, wodurch eher eine bildgebende Diagnostik indiziert werden sollte.

**Leistungsfähigkeit:**

Obwohl das Rauchen weitreichende Folgen am gesamten kardiovaskulären System zeigt, bleibt zu klären, ob Raucher durch eine Modifikation aktueller Leitlinien zur Vorsorge und Diagnose hinsichtlich harter klinischer Endpunkte profitieren würden.

**Empfehlung für die Praxis:**

Raucher sollten aufgrund des deutlich erhöhten kardiovaskulären Risikos zu einer Raucherentwöhnung beraten werden. Hinsichtlich konkreter Krankheitsbilder bedingt das Rauchen keine prinzipielle Modifikation der bildgebenden Abklärung, bei intermediärem Risiko kann aber tendenziell früher zur Bildgebung geraten werden.

## Abstract (en)

**Clinical issue:**

Smoking, which affects the whole cardiovascular system, primarily results in atheromatous plaques with risk of vascular stenosis or aneurysmatic vascular changes with risk of rupture.

**Standard radiological methods:**

Depending on location, sonography provides an initial assessment of alterations. Angiography in combination with computed tomography (CT) and magnetic resonance imaging (MRI) allows further evaluation and, if necessary, therapy planning. In smokers without clinical symptoms or additional risk factors, imaging only because of smoking is not recommended.

**Methodical innovations:**

Recent guidelines of respective pathologies unanimously acknowledge smoking as modifiable risk factor for cardiovascular diseases; therefore, smoking cessation for prevention of secondary acute events is always recommended as the first step. In suspected chronic coronary syndrome, smoking increases clinical probability, which means that diagnostic imaging is often indicated earlier.

**Performance:**

Although smoking causes extensive changes to the entire cardiovascular system, it remains to be evaluated whether smokers might profit from modification of current guidelines regarding prevention and diagnosis in terms of specific clinical events.

**Practical recommendations:**

Due to increased cardiovascular risk, smokers should be advised to stop smoking. Regarding specific diseases, smoking does not fundamentally result in modification of imaging evaluation; however, in intermediate risk patients, further imaging can be recommended earlier in smokers.

Ein lebenslanger Raucher verliert im Vergleich zu Nichtrauchern etwa 10 Jahre an Lebenszeit, und die Hälfte der vermeidbaren Todesfälle bei Rauchern sind auf das Rauchen an sich zurückzuführen [41]. Die Global Burden of Diseases, Injuries and Risk Factors Study (GBD) berichtete bei weltweit insgesamt 7,69 Mio. mit dem Rauchen assoziierten Todesfällen im Jahr 2019 die ischämisch bedingte Herzerkrankung als häufigste Todesursache mit 1,68 Mio. Todesfällen. Nach der chronisch-obstruktiven Lungenerkrankung (COPD) und Malignomen der Trachea, Bronchien und der Lunge ist der Schlaganfall die vierthäufigste Todesursache im Zusammenhang mit dem Rauchen (0,93 Mio. jährliche Todesfälle; [30]). Es sind weltweit also etwa ein Drittel aller mit dem Rauchen assoziierten Todesfälle durch kardiovaskuläre Pathologien bedingt.

In Regionen mit hohem Pro-Kopf-Einkommen stellt die COPD die häufigste tabakrauchassoziierte Todesursache dar, knapp dahinter aber folgen die ischämischen Herzerkrankungen. So waren im Jahr 2019 in der Region Westeuropa, zu der Deutschland, Österreich und die Schweiz zählen, 110.000 tabakrauchassoziierte Todesfälle durch COPD bedingt, 101.000 Todesfälle durch ischämische Herzerkrankungen und 34.800 Todesfälle durch Schlaganfälle [30]. Metaanalysen konnten zudem zeigen, dass Raucher sowohl ein erhöhtes Risiko für das Auftreten als auch für eine Größenzunahme intrakranieller Aneurysmen haben [17, 33]. Ebenfalls konnte eine Assoziation zwischen dem Rauchen und der Prävalenz abdominaler Aortenaneurysmen sowie der peripheren arteriellen Vasosklerose (PAV) gezeigt werden [3, 34]. Diese Daten unterstreichen die Wichtigkeit der Früherkennung kardiovaskulärer Veränderungen bei Rauchern, zu der die Bildgebung einen wichtigen Beitrag leisten kann.

## Mechanismen vaskulärer Schädigung

Häufigste gemeinsame Endstrecke der tabakrauchassoziierten Mechanismen, die das kardiovaskuläre System schädigen, sind einerseits die Arteriosklerose und andererseits aneurysmatische Gefäßveränderungen, wobei u. a. die Koronargefäße, zerebralen Arterien, Nierenarterien, peripheren Arterien und die Aorta betroffen sind. Die Atherogenese wird neben dem Rauchen jedoch auch durch Faktoren wie Diabetes, Hyperlipidämie oder Hypertension beeinflusst.

Die unmittelbarste Folge des Rauchens auf das Gefäßsystem betrifft die endotheliale Funktion. Substanzen im Tabakrauch führen zum einen zu direkter Minderung der Zellviabilität [11]. Zum anderen wird die Fähigkeit endothelialer Zellen, kleine Wunden des Endothels zu reparieren, durch das Rauchen reduziert. Diese Reparaturmechanismen stehen vermutlich mit der Freisetzung von Stickstoffmonoxid (NO) unter laminarem Fluss im Zusammenhang, welche durch Tabakrauch eingeschränkt wird [11]. Des Weiteren wird die Bindungsfähigkeit von Monozyten an Endothelien durch Zigarettenrauch erhöht [11]. Zuletzt kommt es durch das Rauchen zu einer gesteigerten Expression von Adhäsionsmolekülen und Zytokinen durch Endothelzellen, ein Zustand der als proinflammatorischer Phänotyp bezeichnet wird [11]. Interessanterweise wurden die hier genannten Effekte auch für alternative Rauchprodukte (sog. E‑Zigaretten) nachgewiesen, aber mit geringerer Effektgröße als beim konventionellen Rauchen [11].

Durch Endothelschäden und inhibierte Reparaturmechanismen können Lipoproteine in der Gefäßwand abgelagert werden. Monozyten binden an die Endothelzellen, migrieren in das subendotheliale Gewebe und differenzieren im Milieu des proinflammatorischen Phänotyps zu Makrophagen [15]. Oxidationsprodukte der Lipoproteine werden durch die Makrophagen phagozytiert, wodurch sich diese zu Schaumzellen umbilden [15]. Aktivierte T‑Zellen produzieren proinflammatorische Zytokine, wodurch glatte Muskelzellen einwachsen und gemeinsam mit den bisher subendothelial angesammelten Zellen und Lipoproteinen eine atheromatöse Plaque bilden [15].

Rupturiert diese Plaque, wird thrombogenes Material dem Blutfluss exponiert, und eine Thrombusformation mit signifikanter Stenosierung oder Okklusion der betroffenen Gefäße führt zu klinisch akuten Zustandsbildern, wie dem akuten Koronarsyndrom (AKS; [15]).

Auch für die Bildung aneurysmatischer Gefäßveränderungen spielen oxidativer Stress und Veränderungen in der Angiogenese eine Rolle. So konnte an der abdominalen Aorta gezeigt werden, dass bei Rauchern die Genexpression für Proteasen und Faktoren mit Abbau der Matrix als Folge sowie proinflammatorische Zytokine hochreguliert werden [24]. Proliferation und Dedifferenzierung glatter Muskelzellen der Tunica media sowie Abbau extrazellulärer Matrix der Tunica adventitia waren bei Rauchern ebenfalls gesteigert [24].

## Implikationen für Früherkennung und Prävention

Das Rauchen stellt oft einen Risikofaktor unter vielen dar, der über die o. g. Mechanismen am gesamten kardiovaskulären System zu Veränderungen führen kann. Es gibt daher keine Leitlinien für die Behandlung oder Bildgebung von Rauchern per se.

Für die generelle Prävention kardiovaskulärer Erkrankungen empfiehlt die europäische Gesellschaft für Kardiologie (ESC) eine Risikoeinschätzung anhand der Faktoren geografische Region (Schweiz: niedriges Basisrisiko; Deutschland und Österreich: moderates Basisrisiko), Geschlecht, Alter, systolischem Blutdruck, Nicht-HDL-Cholesterin und Rauchen [41]. Das Risiko wird dann entweder über Tabellen oder über Kalkulatoren (z. B. Online, s. Infobox) bestimmt. Die Zuordnung einer bestimmten Risikokategorie zieht nicht automatisch die Empfehlung einer konkreten medikamentösen Therapie oder bildgebenden Abklärung nach sich, eine Raucherentwöhnung wird aber sowohl bei scheinbar gesunden Rauchern als auch bei Rauchern mit bekannter Koronarsklerose immer als erster Schritt zur Senkung des kardiovaskulären Risikos empfohlen (Tab. 1; [41]). Zur weiteren Entscheidung, ob eine – ggf. medikamentöse – Therapie zum Erreichen bestimmter Zielwerte hinsichtlich Blutdruck, Nicht-HDL-Cholesterin oder Blutzucker eingeleitet werden sollte, können bestimmte Modifikatoren insbesondere bei Patienten mit einem Risiko an der Grenze zwischen zwei Risikokategorien herangezogen werden [41]. Unter anderem kann die Bildgebung zur Entscheidungsfindung beitragen. Patienten mit einem koronaren Kalzium-Score über dem alters- und geschlechtsadaptierten Durchschnitt sollten unter Berücksichtigung sonstiger Risikofaktoren und -modifikatoren eher einer intensiveren Therapie zugeführt werden [41]. Die sonographische Messung der generellen Intima-media-Dicke wird nicht zur Risikostratifizierung empfohlen, da der Parameter keine Verbesserung der Risikoeinschätzung bedingt [8]. Im Gegensatz dazu sind fokale Plaques der Karotis mit kardiovaskulären Ereignissen assoziiert. Da die Datenlage hier weniger umfassend ist, lautet die Empfehlung aber, die Karotissonographie als Alternative zum Kalzium-Score zu sehen, wenn die kardiale Computertomographie (CT) nicht durchführbar oder nicht beurteilbar ist [41].

**Table 1:** 

Alter	Frauen	Männer
90+	0,8–0,9	0,5–0,7
85–89	1,6–1,9	0,7–1,1
80–84	2,0–2,5	1,2–1,5
75–79	2,6–3,0	1,6–2,0
70–74	3,0–3,4	2,0–2,5
65–69	3,3–3,9	2,4–2,9
60–64	3,6–4,3	2,7–3,4
55–59	3,8–4,6	2,8–3,8
50–54	3,9–4,9	2,9–4,2
45–49	4,1–5,1	3,1–4,4
40–44	4,1–5,2	3,2–4,5

Geschätzte zusätzliche Anzahl an Jahren ohne einen myokardialen Infarkt oder Schlaganfall, wenn eine Raucherentwöhnung zu einem gewissen Lebensalter durchgeführt wird, aufgeschlüsselt nach Geschlecht. Die Werte innerhalb der Alters- und Geschlechtskategorie hängen zusätzlich noch vom systolischen Blutdruck und dem Nicht-HDL-Cholesterin ab. Für eine detailliertere Tabelle siehe [41]

## Kardiale raucherassoziierte Veränderungen

### Chronisches Koronarsyndrom

Das Risiko, ein chronisches Koronarsyndrom (CKS) zu entwickeln ist beim Rauchen von 20 Zigaretten täglich im Vergleich zu Nichtrauchern etwa doppelt so hoch. Obwohl eine gewisse Dosisabhängigkeit besteht, ist das Risiko bereits beim Rauchen von nur einer Zigarette pro Tag 1,5fach erhöht [12]. In einer Registerstudie, in welcher weltweit in 45 Ländern über 32.000 Patienten mit CKS eingeschlossen wurden, wurde das Rauchen neben anderen Faktoren als unabhängiger Prädiktor für das Auftreten der primären Studienendpunkte kardiovaskulärer Tod und nichttödlicher Myokardinfarkt innerhalb von 5 Jahren identifiziert (Hazard-Ratio [HR] 1,74). Ehemalige Raucher hatten immerhin noch ein 1,29fach erhöhtes Risiko [35].

Die koronare Herzerkrankung (KHK) ist definiert als eine Akkumulation von arteriosklerotischen Plaques in epikardialen Gefäßen [20]. Kommt es zu einer Plaqueruptur mit – wie in der Einleitung erwähnt – thrombotischer Obliteration, resultiert das AKS mit dem Risiko myokardialer Infarzierung bis hin zum Tod. Um dem Übergang aus einem chronischen in ein akutes Koronarsyndrom entgegenzuwirken, empfehlen die ESC-Leitlinien aus 2019 eine Kombination aus Lebensstilmodifikation, pharmakologischer Therapie und invasiver Intervention, wobei die Risikostratifizierung anhand diagnostischer Tests erfolgt [20].

**Figure 1:**
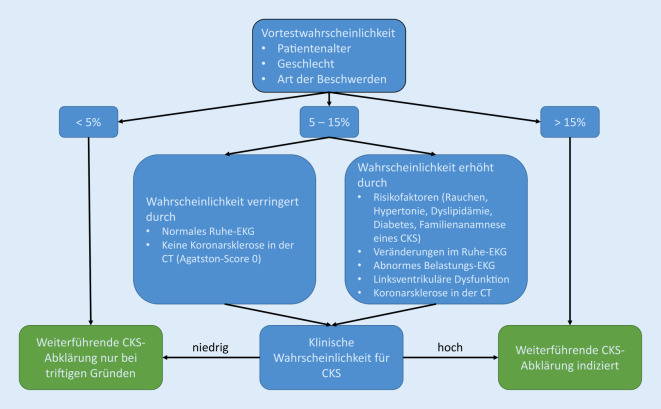

Die Indikation zur weiteren bildgebenden Diagnose sollte je nach klinischer Wahrscheinlichkeit für das Vorliegen einer koronaren Gefäßerkrankung erfolgen. Das Rauchen ist dabei ein Faktor, der bei intermediärer Vortestwahrscheinlichkeit die klinische Wahrscheinlichkeit für das Vorliegen eines CKS erhöht und damit zur Entscheidung für eine weiterführende nichtinvasive Abklärung beitragen kann (Abb. 1; [20]). Eine weiterführende Übersicht über die Empfehlungen der ESC hinsichtlich Bildgebung bei Verdacht auf ein CKS ist in *Der Radiologe *Volume 60, Ausgabe 12 zu finden [13].

**Figure 2:**
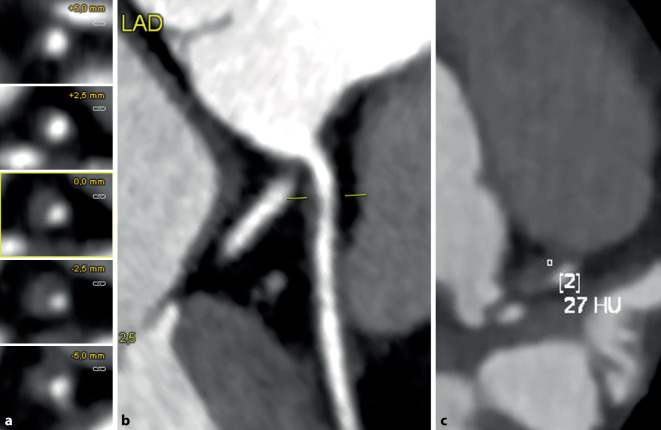

CT-morphologisch kann bei Vorliegen eines CKS neben der Quantifizierung einer Koronarstenose zur Einschätzung des Risikos für ein AKS auch die Vulnerabilität der koronaren Plaque beurteilt werden. Eine Plaque wird CT-morphologisch als Hochrisikoplaque bezeichnet, wenn mindestens 2 von 4 möglichen CT-Charakteristika (positives Remodeling, geringe Dichte < 30 HE, punktförmige Kalzifizierungen, *Serviettenring-Zeichen*) vorliegen [7]. Rauchen konnte dabei neben Übergewicht als Faktor identifiziert werden, der mit dem Vorliegen von Hochrisikokriterien in der CT assoziiert ist (Abb. 2; [32]).

**Figure 3:**
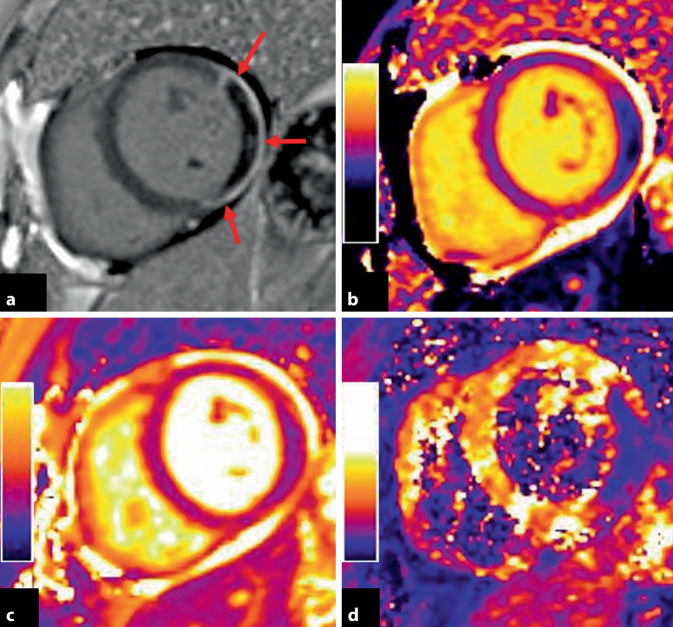

Als *Raucher-Paradoxon* wurde die Beobachtung bezeichnet, dass Raucher im Fall eines AKS im Vergleich zu Nichtrauchern ein höheres Überleben und weniger kardiovaskuläre Folgeereignisse zeigten [31]. Neuere Studien nach primärer perkutaner Koronarintervention konnten aber zeigen, dass der scheinbare Überlebensvorteil durch das jüngere Alter von Rauchern zum Zeitpunkt des Infarkts und damit eine geringere Zahl an Komorbiditäten dieser Patienten erklärbar ist. Darüber hinaus zeigten sich die Infarktgröße sowie die Größe des Areals, das durch die Therapie vor einer endgültigen Infarzierung bewahrt werden konnte, unabhängig vom Raucherstatus. Für die mikrovaskuläre Obstruktion im akuten Myokardinfarkt, die mit einer signifikant schlechteren Prognose assoziiert ist, wurde teilweise eine größere Ausdehnung bei Rauchern beschrieben (Abb. 3; [28, 29]). Vor allem im Hinblick auf das höhere Risiko eines AKS sollte das Rauchen insgesamt weiterhin als Risikofaktor sowohl für das chronische als auch akute Koronarsyndrom und klinische Endpunkte wie Tod oder Reinfarkt im Zusammenhang mit dem AKS gesehen werden.

### Herzklappen

Rauchen wirkt sich auch auf die Herzklappen nachteilig aus und führt vorwiegend zu Stenosen durch Kalzifizierungen. So konnte gezeigt werden, dass Aortenklappenstenosen bei Rauchern signifikant häufiger zu finden sind. Nach einer Raucherentwöhnung nähert sich das Risiko langsam wieder dem von Nichtrauchern an mit einem vergleichbaren Risiko 10 Jahre nach der Entwöhnung [22].

**Figure 4:**
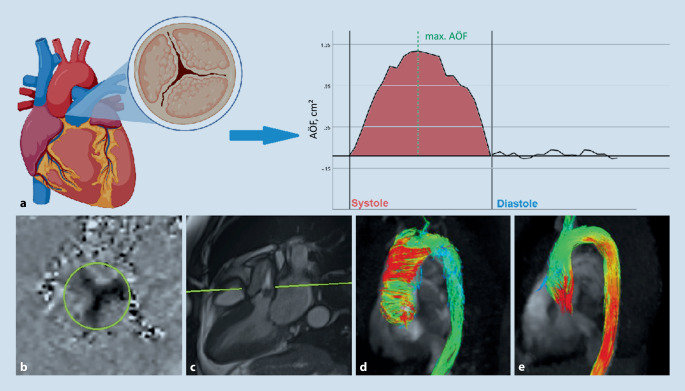

Obwohl Verkalkungen der Aortenklappe CT-morphologisch besser abzugrenzen sind, bietet die MRT die Möglichkeit, in einer Untersuchung zusätzliche Informationen zu gewinnen, wie ventrikuläre Volumina, eventuelle Myokardfibrosierung mittels nativem T1-Mapping und Bestimmung des Extrazellularvolumens (s. auch [26]) oder ein eventuelles fokal myokardiales Late-Gadolinium-Enhancement, welche bei interventionspflichtiger schwerer Aortenstenose einen prognostischen Wert für das klinische Ergebnis gezeigt haben [38]. Darüber hinaus erlaubt die Flussmessung mittels zweidimensionaler Phasenkontrasttechnik eine Einschätzung des maximalen Druckgradienten über der Aortenklappe und der Klappenöffnungsfläche [4, 25, 39]. Vierdimensionale Flussmessungen (d. h. zeitlich aufgelöste, EKG-getriggerte Aufnahmen nicht nur unidirektionaler, sondern aller 3 Geschwindigkeitskomponenten) erlauben eine wesentlich detailliertere Analyse der makroskopischen Strömungsmechanik und mittels dezidierter Post-processing-Software können Blutströmungen als Geschwindigkeitsvektoren, Stromlinien (Tangentialkurven an Geschwindigkeitsvektoren zu einer bestimmten Zeit) oder Teilchenpfade visualisiert, sowie die Ermittlung von Flüssen durch beliebige Querschnittsflächen ermöglicht werden (Abb. 4; [9]).

## Raucherassoziierte Veränderungen am peripheren Gefäßsystem

### Periphere arterielle Vasosklerose

Auch für die PAV konnte das Rauchen als Risikofaktor identifiziert werden (HR 3,43 für aktive Raucher in Ländern mit hohem Pro-Kopf-Einkommen; [34]). Unter dem Begriff der PAV werden arteriosklerotische Veränderungen extrakranieller gehirnversorgender Arterien, mesenterialer Arterien, renaler Arterien und der Extremitätenarterien subsummiert [1]. Bezüglich Bildgebung wird auch hier die Sonographie als niederschwellig verfügbare und kostengünstige Modalität empfohlen, mit der beispielsweise Plaques der A. carotis morphologisch als auch im Hinblick auf Auswirkungen auf den Blutfluss evaluiert werden können [1]. Die CT-Angiographie bietet wiederum eine Darstellung des Gefäßsystems in hoher Auflösung mit kurzer Untersuchungszeit und ermöglicht so z. B. eine Therapieplanung. Die Notwendigkeit der Gabe jodhaltiger Kontrastmittel schränkt aber die Anwendbarkeit bei Patienten mit eingeschränkter Nierenfunktion ein [1]. Die MR-Angiographie kann als Alternative zur CT-Untersuchung eingesetzt werden [1]. In einer rezenten Metaanalyse konnte die hohe Sensitivität (0,88) und Spezifität (0,94) der kontrastmittellosen Quiescent-interval-single-shot(QISS)-MR-Angiographie zur Evaluierung der PAV an der unteren Extremität gezeigt werden [40]. Sofern keine Kontraindikation für eine MRT-Untersuchung vorliegt, bietet sich die kontrastmittellose MR-Angiographie insbesondere bei Patienten mit eingeschränkter Nierenfunktion zur Diagnostik an. Die invasive digitale Subtraktionsangiographie (DSA) spielt noch für die PAV unterhalb des Knies eine diagnostische Rolle, wurde aber ansonsten weitgehend durch die o. g. Modalitäten abgelöst [1].

Hinsichtlich des Rauchens empfiehlt die ESC keine Modifikation ihrer Leitlinien für Diagnostik oder Therapie der PAV. Die Raucherentwöhnung wird aber bei allen Manifestationen der PAV dringend zur generellen Reduktion des kardiovaskulären Risikos empfohlen (Klasse I, Level B; [1]).

### Schlaganfall

In einer großen Metaanalyse konnte eine Assoziation von aktivem Rauchen und Schlaganfällen (OR 1,47) mit Sichtbarkeit in der MRT (hohe Signalintensität in der T2-Gewichtung oder FLAIR-Gewichtung [„fluid attenuated inversion recovery“]) hergestellt werden [5]. Insbesondere für subarachnoidale Blutungen ist das Rauchen der größte modifizierbare Einflussfaktor mit einem 2‑ bis 4fach erhöhten Risiko bei Rauchern [19].

Die Leitlinien der Amerikanischen Herzgesellschaft (AHA) gemeinsam mit der Amerikanischen Schlaganfallgesellschaft (ASA) geben keine Empfehlung zur Bildgebung in der Primärprävention von Schlaganfällen. Es gibt aber eine ganz klare Empfehlung, Patienten zu einer Raucherentwöhnung zu raten bzw. passives Rauchen zu vermeiden, um das Risiko eines Schlaganfalls wieder nahezu auf das Niveau eines lebenslangen Nichtrauchers zu bringen [18].

### Intrakranielle Aneurysmen

Rauchen stellt einen Risikofaktor für das Auftreten intrakranieller Aneurysmen dar, weshalb sowohl die AHA/ASA-Leitlinien von 2015 als auch die Leitlinien der Europäischen Schlaganfallgesellschaft (ESO) von 2013 bei Patienten mit unrupturiertem intrakraniellem Aneurysma (UIA) eine Raucherentwöhnung empfehlen (Klasse I, Evidenzlevel B; [36, 37]). Die International Study of Unruptured Intracranial Aneurysms (ISUIA) berichtete in einer prospektiven Kohorte einen Anteil von insgesamt 77 % aktueller oder ehemaliger Raucher bei Patienten mit UIA [43]. In einer kontrollierten multizentrischen Studie konnte gezeigt werden, dass bei Frauen zwischen 30 und 60 Jahren das Rauchen ein knapp 4fach höheres Risiko für UIA bedingt, wobei das Risiko durch eine gleichzeitig vorliegende Hypertonie auf das 6Fache erhöht wird [27]. Auch in der Therapieentscheidung sollte nach ESO-Leitlinien das Rauchen als Faktor mit Erhöhung des Rupturrisikos eines UIA beachtet werden [36]. Für Aneurysmata über 7 mm Durchmesser konnte das Rauchen als Risikofaktor für eine Ruptur identifiziert werden, ein Zusammenhang, der bei einem Durchmesser unter 7 mm aber nicht nachgewiesen werden konnte [21, 23].

Eine Empfehlung zu Screening-Untersuchungen, entweder mittels CT- oder MR-Angiographie wird für Patienten mit 2 oder mehr Angehörigen mit intrakraniellen Aneurysmen oder stattgehabter subarachnoidaler Blutung ausgesprochen, insbesondere beim Vorliegen der zusätzlichen Risikofaktoren Rauchen, Hypertension oder weibliches Geschlecht (Klasse I, Evidenzlevel B; [36, 37]). Eine Empfehlung zum Screening asymptomatischer Patienten ohne familiäre Belastung oder sonstige erbliche Faktoren lediglich aufgrund des Rauchens gibt es mangels Daten zurzeit nicht.

Zur bildgebenden Diagnose werden die MR/CT-Angiographie und die digitale Subtraktionsangiographie (DSA) empfohlen. Aufgrund der hohen räumlichen Auflösung und der unmittelbaren Möglichkeit zur interventionellen Behandlung eines Aneurysmas stellt die DSA weiterhin den diagnostischen Goldstandard dar. Verbesserungen in der bildgebenden Technik und der invasive Charakter der DSA haben aber dazu geführt, dass die MR/CT-Angiographie zunehmend als primäre bildgebende Modalitäten eingesetzt werden [37]. In Tab. 2 ist die diagnostische Wertigkeit der MR/CT-Angiographie im Vergleich zur Goldstandard-Untersuchung dargestellt.

**Table 2:** 

	3D-TOF-MRA	CTA
Sensitivität	0,89 (0,82–0,94)	0,84 (0,81–0,86)
Spezifität	0,94 (0,86–0,97)	0,85 (0,79–0,89)
LR+	13,79 (5,92–32,12)	4,09 (2,45–6,81)
LR−	0,11 (0,07–0,19)	0,18 (0,11–0,28)
AUC	0,96	0,90

*AUC* area under the curve, *CTA* CT-Angiographie, *LR* Likelyhood-Ratio, *MRA* MR-Angiographie, *TOF* time of flight

Hinsichtlich Morphologie und Lokalisation haben Raucher häufiger Aneurysmata des Basilariskopfes (HR 6,26), multiple Aneurysmata (HR 2,15), einen größeren Durchmesser des abführenden Gefäßes (HR 3,13) und eine größere maximale Höhe des Aneurysmas in Relation zum Durchmesser des Trägergefäßes (HR 1,78; [16]).

Entscheidet man sich für ein konservatives Management eines UIA, sollte dieses in regelmäßigen Intervallen mittels nichtinvasiver Bildgebung kontrolliert werden (Klasse I, Evidenzlevel B). Die Evidenz bezüglich des optimalen Intervalls ist weniger stark, empfohlen wird aber ein Abstand von 6 bis 12 Monaten für die erste Folgeuntersuchung, danach kann eine jährliche oder 2‑jährliche Untersuchung erfolgen [37]. Eine klare Empfehlung zur Modifikation der Kontrollintervalle aufgrund des Rauchens gibt es nicht. Nach endovaskulärer Therapie sind Folgeuntersuchungen zur Sicherstellung eines suffizienten Behandlungsergebnisses indiziert, wobei eine Strategie mit primärer Kontrolle mittels MR-Angiographie und ggf. weiterführender DSA bei Hinweisen auf eine Reperfusion sinnvoll erscheint [37]. Eine Raucheranamnese ging in einer retrospektiven Analyse mit einer knapp 3fach erhöhten Rate für eine Revaskularisierung eines primär suffizient okkludierten UIA einher, wobei kein relevanter Unterschied zwischen aktiven und ehemaligen Rauchern bestand [10].

### Abdominelles Aortenaneurysma

Aus dem bisher Gesagten geht der große Einfluss des Rauchens auf das gesamte kardiovaskuläre System hervor. Betrachtet man den Einfluss des Rauchens auf die Entwicklung abdomineller Aortenaneurysmen (AAA), so ist das Risiko, ein solches zu entwickeln, bei Rauchern im Vergleich zu Nichtrauchern noch einmal deutlich höher (HR 4,79) als für die Entwicklung eines isolierten CKS, ischämischen Schlaganfalls oder einer PAV (HR 1,97; [2]).

Die aktuellen Leitlinien der Europäischen Gesellschaft für Gefäßchirurgie empfehlen generell für Männer über 65 Jahre ein einmaliges sonographisches Screening zur Erkennung eines eventuellen AAA, während für Frauen keine Empfehlung zum Screening gegeben wird. Obwohl Schätzungen zufolge etwa 75 % der AAA hauptsächlich auf das Rauchen zurückzuführen sind, wird keine Empfehlung für ein frühzeitiges Screening bei männlichen Rauchern oder ein generelles Screening bei weiblichen Rauchern gegeben [42]. Zur Bildgebung wird primär die Sonographie empfohlen. Zeigt sich sonographisch ein maximaler Durchmesser des Aneurysmas um den oder über dem Grenzwert für eine operative Rekonstruktion, ist eine kontrastmittelgestützte CT-Angiographie mit zusätzlicher Darstellung der thorakalen Aorta empfohlen. Die MR-Angiographie kann als Alternative zur CT-Angiographie eingesetzt werden, insbesondere um dem Patienten die Strahlendosis zu ersparen, wenn eine wiederholte Bildgebung notwendig ist. Das Rauchen spielt für die Wahl der bildgebenden Methode keine Rolle [42].

#### Infobox

Online-Rechner zur Einschätzung des kardiovaskulären Risikos:

https://u-prevent.com/calculators

## Fazit für die Praxis

- Rauchen stellt einen Risikofaktor für die Entstehung kardiovaskulärer Pathologien am gesamten Körper dar.
- Meist handelt es sich dabei um arteriosklerotische Veränderungen mit dem Risiko der signifikanten Gefäßstenose/Okklusion oder aneurysmatische Veränderungen mit Rupturrisiko.
- Es gibt keine spezifischen Empfehlungen für ein Screening lediglich aufgrund des Rauchens.
- Rauchen sollte aber immer als Faktor für die Entscheidung zur bildgebenden Diagnostik bei klinischem Verdacht auf kardiovaskuläre Veränderungen oder für die Therapieentscheidung miteinbezogen werden.
- Die Wahl der bildgebenden Modalität richtet sich nach Körperregion, klinischer Wahrscheinlichkeit für das Vorliegen kardiovaskulärer Veränderungen und Indikation zur Bildgebung.
- Generell sollten die Patienten hinsichtlich einer Raucherentwöhnung beraten werden, da das kardiovaskuläre Risiko keine lineare Dosisabhängigkeit zeigt und bereits eine einzelne Zigarette pro Tag ein erheblich erhöhtes Risiko mit sich bringt.

## Abkürzungen

AAA: Abdominelles Aortenaneurysma
AKS: Akutes Koronarsyndrom
CKS: Chronisches Koronarsyndrom
CT: Computertomographie
DSA: Digitale Subtraktionsangiografie
ESC: European Society of Cardiology (Europäische Gesellschaft für Kardiologie)
HE: Houndsfield-Einheiten
MRT: Magnetresonanztomographie
PAV: Periphere arterielle Vasosklerose
UIA: Unrupturiertes intrakranielles Aneurysma
